# Community-based transport system in Shinyanga, Tanzania: A local innovation averting delays to access health care for maternal emergencies

**DOI:** 10.1371/journal.pgph.0001487

**Published:** 2023-08-02

**Authors:** Castory Munishi, Gilbert Mateshi, Linda B. Mlunde, Belinda J. Njiro, Jackline E. Ngowi, James T. Kengia, Ntuli A. Kapologwe, Linda Deng, Alice Timbrell, Wilson Kitinya, Andrea B. Pembe, Bruno F. Sunguya

**Affiliations:** 1 Muhimbili University of Health and Allied Sciences, Dar es Salaam, Tanzania; 2 President’s Office Regional Administration and Local Government, Dodoma, Tanzania; 3 Touch Foundation, Mwanza, Tanzania; Jhpiego, UNITED STATES

## Abstract

In achieving the sustainable development goal 3.1, Tanzania needs substantial investment to address the three delays which responsible for most of maternal deaths. To this end, the government of Tanzania piloted a community-based emergency transport intervention to address the second delay through m-mama program. This study examined secondary data to determine the cost-effectiveness of this intervention in comparison to the standard ambulance system alone. The m-mama program was implemented in six councils of Shinyanga region. The m-mama program data analyzed included costs of referral services using the Emergency Transportation System (EmTS) compared with the standard ambulance system. Analysis was conducted using Microsoft Excel, whose data was fed into a TreeAge Pro Healthcare 2022 model. The cost and effectiveness data were discounted at 5% to make a fair comparison between the two systems. During m-mama program implementation a total of 989 referrals were completed. Of them, 30.1% used the standard referral system using ambulance, while 69.9% used the EmTS. The Emergency transport system costed USD 170.4 per a completed referral compared to USD 472 per one complete referral using ambulance system alone. The introduction of m-mama emergency transportation system is more cost effective compared to standard ambulance system alone in the context of Shinyanga region. Scaling up of similar intervention to other regions with similar context and burden of maternal mortality may save cost of otherwise normal emergency ambulance system. Through lessons learned while scaling up, the intervention may be improved and tailored to local challenges and further improve its effectiveness.

## Introduction

The first target of Sustainable Development Goal (SDG) number three aims to reduce global maternal mortality ratio (MMR) to less than 70 per 100,000 live births by 2030 [1]. Few years to the SDG’s deadline, countries in sub-Sahara are far behind and need innovative approaches to meet this target [2]. Tanzania has one of the highest MMR in the world [3, 4]. Effective interventions to address the burden of maternal deaths are known, however, sustainability of such interventions requires a careful tailoring to suit the local context [5].

Like in other contexts, causes of maternal and perinatal deaths in Tanzania fit in a three-delay model [6]. The first is the delay in making the decision to seek care when experiencing an obstetric emergency. The second is the delay in reaching an appropriate obstetric facility once the decision has been made to go. The third, is the delay in receiving adequate and appropriate care once the facility has been reached [6]. These delays do not operate independently, and in some cases, one may have an influence on another [7]. For example, expectations of transport delays or of low-quality care at the nearest facility or the distance to reach the facility with no appropriate means to reach there, influence the initial decision to seek care [8].

Community level interventions have an important role to play in addressing the burden of maternal mortality [9] Such interventions include training of community health workers to recognize danger signs in pregnancy, provision of nutritional supplements to pregnant women, increasing community awareness of danger signs of pregnancy, and need to seek early care [10, 11]. Available evidence supports the use of community-based interventions in addressing the three delays [12].

The m-Mama program is one of the community-based innovation that was implemented in Shinyanga region, Tanzania to reduce the burden of maternal mortality [13]. The program was designed and implemented by the government of Tanzania in collaboration with Touch Foundation, Pathfinder International, and Vodafone foundation in Shinyanga region, Tanzania [13]. Through the program, an innovative Emergency Transportation System (EmTS) utilizing digital technology to remotely triage patients was developed and implemented [14]. After triage through the digital platform, an ambulance or a community taxi is dispatched to transfer the patient to a designated health facility, suitable to address the condition. The community taxis involved in the program were driven by local drivers who were members of the community trained to operate as back-up emergency transport when standard ambulances were not available. In the standard ambulance system, government employed drivers were deployed to the health facilities in the region.

While directly addressing transportation, the program also included health system strengthening and community education initiatives [13]. The program is initiated and worked with Community Care Groups to encourage health seeking behavior and ensure women receive high-quality care upon reaching the appropriate health facility. The end line evaluation of the m-mama pilot program indicated a significant reduction of maternal deaths in Shinyanga region from 60 in 2015 to 15 in 2021 [15]. This program could have a significant contribution among other evidence-based interventions to improve health system and maternal health specific programs in the region. This study therefore aimed to determine the cost-effectiveness of this intervention compared with the standard emergency referral system for scaling up nationally.

## Methods

### Study design and settings

This cost-effectiveness analysis was part of the end line m-mama program evaluation and used secondary data from the program documents. The analysis aimed to compare the cost-effectiveness of the EmTs against the standard system which uses ambulances only to provide referral services for pregnant women in the Shinyanga region.

Shinyanga region has a population of about 1.9 million people, with about 92,000 births annually [16]. Of them, 83.4% live in rural areas [16]. The region has a total land area of 18,901 km^2^ with an average population density of 130 people per km^2^. Administratively, Shinyanga region has six councils namely Kishapu District Council (DC), Kahama Town Council (TC), Msalala DC, Shinyanga DC, Shinyanga Municipal Council (MC) and Ushetu DC. At the council level, Shinyanga DC has the highest population with 417,000 people and the highest birth rate (20,100 births/year) **Table 1**. Shinyanga MC has the highest population density 291 people per km^2^ owing to its urbanicity [16].

**Table 1:** Summary of council demographics and geography.

Council	Population	Estimated Births annual births	Population Density per km^2^ by District
**Kishapu DC**	344,000	16,400	65
**Kahama TC**	308,000	14,600	178
**Msalala DC**	313,000	15,100	78
**Shinyanga DC**	417,000	20,100	94
**Shinyanga MC**	209,000	9,700	291
**Ushetu DC**	342,000	16,400	78
**Total**	1,933,000	92,300	130

The EmTS is comprised of normal ambulance services and a community taxi when an ambulance is not available. For this analysis, the EmTS was compared with the standard model of referral, a normal ambulance transport system. The EmTS was used in the Shinyanga region to provide an improved means of transport for pregnant women and their newborns during times of emergencies in six councils.

During program implementation, the region had a total of 19 ambulances, ranging between two to four ambulances per council. Kahama TC and Msalala DC each had two ambulances, Ushetu DC had three while Shinyanga MC, Shinyanga DC and Kishapu each had four ambulances. Similarly, the number of designated community taxi drivers ranged from eight to 22 per council. Kishapu DC had 22 community taxi drivers, while Kahama TC and Msalala DC each with eight community taxi drivers (**Table 2**).

**Table 2:** Ambulance and drivers distribution.

Council	Number of Ambulances	Ambulances per population	Ambulances per km^2^	Number of Community drivers
**Kishapu DC**	4	1 per 86,000 people	1 per 1050	22
**Kahama TC**	2	1 per 154,000 people	1 per 760	8
**Msalala DC**	2	1 per 156,500 people	1 per 1,820	8
**Shinyanga DC**	4	1 per 104,300 people	1 per 1050	13
**Shinyanga MC**	4	1 per 52,300 people	1 per 330	10
**Ushetu DC**	3	1 per 144,000	1 per 1,770	14

The m-mama program enrolled dispatchers from the existing council health facilities to work at the dispatch center located at the council level. The enrollees were mostly nurses and nurse assistants. The dispatchers received a three-day training on remote triaging, handling maternal emergencies, and standard operating procedures for the dispatch center operations. A toll-free number in case of emergency to the dispatch center was also introduced to offer a 24/7 access to emergency transportation. Upon receiving an emergency call, a trained dispatcher would remotely triage the patient after an initial assessment through a series of pre-defined questions. Supported by a decision-making application running on a tablet, the dispatcher would enter the information received, and the application would trigger a set of sequential logical questions. The application would then indicate whether the patient needs to be transferred to a designated health facility based on her condition. This process would take an average of two minutes. The dispatch application displayed the available drivers based on proximity with where the emergency was happening.

The dispatch application is integrated with a mobile payment system ensuring a fast and responsive payment for a community driver who completed the referral transport action. The community taxi drivers were paid based on the total distance covered at an average of 1,000 Tanzania Shilling ($43.5 cents) per km. To ensure sustainability, the program followed a progressive transition on its funding approach, at the start (first stage January to December 2019) the program financed 80% of the transportation costs, on the second stage (January to December 2020) the program financed 50% and the government 50%, and on third stage from January 2021 the government of Tanzania financed 100% of the EmTS.

### Target population

The emergency transportation system was built to facilitate transport of women during emergencies from the community to facilities, or from one facility to another in Shinyanga Region.

### Source of data

Cost data for the emergency transportation system and the use of ambulances were provided by the m-Mama program. The data covered the costs incurred in the final year spanning July 1^st^, 2020, to June 30^th^, 2021.

### Variables and analysis context

The key variables and parameters in the cost effectiveness analysis were the cost of completed referrals and the number of referrals completed by both the ambulance and the community taxis. The outcome measures of interest were the cost, effectiveness, and the cost effectiveness of the interventions used for completion of emergency referrals. We took a provider’s perspective in estimating the cost-effectiveness whereby costs incurred by the referral providers during the provision of EmTS were compared. During the analysis, the cost of the emergency transportation system using community taxis and ambulances was compared against the standard system which used ambulances only. Completed referrals were used as a proxy for effectiveness based on the available program data.

The evaluated costs and outcomes covered July 1^st^, 2020, to June 30^th^ 2021. A 5% discount rate used was provided by the Bank of Tanzania (BoT). The costs in the analysis were reported in Tanzanian shillings and they were reported for July 2020/June 2021 a twelve months period and these were further converted to USD using the BoT exchange rates at the time of implementation.

The analyses conducted had the following four assumptions. First, the EmTS complements the standard of care (Ambulance only and therefore EmTS comprised of the standard of care plus the community taxi services. Second, the cost of service included all costs incurred by the provider in referral services excluding fuel cost. The final cost was therefore a total of services and diesel costs. We assumed that the training costs for dispatchers and other recurrent costs remained the same as these costs were incurred both in the standard referral system (Ambulances only) and the new intervention (Ambulance and community taxis) and hence they were not included in this analysis. Third, all the referrals were completed during the time the costs were incurred (July 2020/June 2021). Fourth, all completed referrals were effective referrals and the decision tree for both strategies was not branched into effective and ineffective referrals.

The following calculations were made during the analysis: The Incremental cost (Incr Cost) was calculated as the difference in cost between the EmTS and the standard referral system; Incremental Effectiveness (Incr Eff) was calculated as the difference between the effectiveness of the EmTS and the standard referral system; Cost-effectiveness was calculated as the ratio of cost and effectiveness for each intervention, and the Incremental cost-effectiveness ratio (ICER) was calculated as the ratio of incremental cost to incremental effectiveness. This represented the additional cost per unit of effectiveness to switch to a more effective strategy.

**Fig 1:**
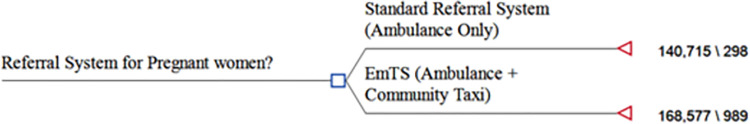
The decision tree comparing the cost and effectiveness of the standard referral system and the emergency transport system.

Data was cleaned using Microsoft excel 2016 version. The total costs were then calculated and summarized in tabular format and then inputted into TreeAge Pro Healthcare 2022 software using a decision tree model for analysis of costs and effectiveness (**Fig 1**). The TreeAge Pro was used because of its ease to use in estimating the cost effectiveness and availability [17]. The decision tree model generated a cost-effectiveness ranking report. Finally, the costs of both ambulances and the new EmTS were discounted at 5% as to make fair comparisons as costs and outcomes did not occur at the same time [18].

### Ethical consideration

Ethical approval for this evaluation was granted by the Muhimbili University of Health and Allied Sciences Institutional Review Board (MUHAS-REC-11-2021-885). Permission to collect data from Shinyanga region was granted by the President’s Office Regional and Local Government Authority (PO-RALG) and the office of the Regional Medical Officer (RMO) in Shinyanga. Written informed consent was obtained from participants before data collection. Privacy and confidentiality were maintained. The data obtained from this program evaluation were kept as strictly confidential, were accessible to only the named investigators and have been stored on password-protected computers.

## Results

### General characteristics of emergency referrals

Data on costs and completed referrals for the Shinyanga region, from July 2020 to June 2021 (FY 2020/21) were used for this cost-effectiveness analysis. During this period, the six councils completed a total of 989 referrals. These were completed using both the standard ambulance referral services and the community taxis. Out of all referrals, 30.1% were completed using the standard referral system using ambulance services while the majority 69.9% were completed by the use community taxis; an intervention that was introduced along the standard referral system.

In Shinyanga municipal council, the majority (90%) of the referrals were carried out using ambulances. Msalala DC, Kishapu DC, and Shinyanga DC which are more rural areas had a higher proportion of trips completed through community taxis (**Table 3)**.

**Table 3:** Total number of referrals completed.

Council	Ambulance referrals, n (%)	Community taxi referrals, n (%)
**Kishapu DC**	51 (17.6)	239 (82.4)
**Kahama TC**	23 (43.4)	30 (56.6)
**Msalala DC**	15 (13.3)	98 (86.7)
**Shinyanga DC**	64 (19.3)	267 (80.7)
**Shinyanga MC**	117 (90.0)	13 (10.0)
**Ushetu DC**	28 (38.9)	44 (61.1)
**Total**	**298 (30.1)**	**691 (69.9)**

### Total cost of emergency referrals

A total of TZS 389.4 million was spent during FY2020/21 among six councils of Shinyanga region while completing all the referrals. Most of the cost was incurred on ambulance trips although there were just a few ambulances (**Table 4)**. The standard ambulance referrals represented 83% of the total cost used, with Kishapu DC covering most of the expenditures at 29% and Shinyanga MC being the least at 7%. For community taxi referrals, the highest cost came from Kishapu DC (40%), and the least was from Shinyanga MC (1%). Table 2 Shows the total cost of handling referrals using by using ambulances alone and by the new intervention.

**Table 4:** Total cost of referrals completed for FY2020/21.

Council	Total Cost of Ambulance referrals (TZS)	Total Cost of Community taxi referrals (TZS)
**Kishapu**	94,000,000	26,000,000
**Kahama**	73,000,000	2,440,000
**Msalala**	40,000,000	8,186,000
**Shinyanga DC**	36,000,000	23,758,000
**Shinyanga MC**	23,000,000	374,000
**Ushetu**	59,000,000	3,594,000
**Total**	**325,000,000**	**64,352,000**

**Table 5** shows mean costs for operating ambulances and EmTS. For both ambulances and EmTS, Kahama TC had a mean costs 3,173,913 TZS for ambulance and 1,423,396 TZS for EmTS presenting the highest mean operational costs. This was followed by Ushetu DC which had means of 2,107,143 TZS and 869,361 TZS for ambulances and EmTS respectively. Shinyanga MC had the lowest mean costs for both ambulances and EmTS. Except for Shinyanga MC, all other councils had the mean costs for operating ambulances between 2 to 5 times that of using EmTS.

**Table 5:** Mean of completed referrals for FY 2020/2021.

Council	Mean Cost TZS	
	Ambulance	Emergency Transport system
**Kishapu DC**	1,843,137	413,793
**Kahama TC**	3,173,913	1,423,396
**Msalala DC**	2,666,667	426,425
**Shinyanga DC**	562,500	180,538
**Shinyanga MC**	196,581	179,800
**Ushetu DC**	2,107,143	869,361

### Cost-effectiveness

For cost-effectiveness calculations, all referrals were considered completed referrals. Table 6 shows the undiscounted cost and effectiveness between the standard intervention (ambulance only) and the new intervention (ambulance plus community taxis for FY2020/21).

**Table 6:** Summary of undiscounted cost and effectiveness for Shinyanga region.

Interventions/Strategy	Costs in TZS	Cost in USD**	Number of referrals
**Standard (Ambulance only)**	325,000,000	140,715	298
**EmTS (Ambulance+ Community Taxi)**	389,352,000	168,578	989

*Note: the exchange rate used = 2,309.63 (BoT, 20 Feb 2022), **USD = United States Dollar

Using the TreeAge Pro Healthcare 2022, the annual incremental cost and effectiveness between the standard and the new intervention were $27,862 and $ 657 respectively and an incremental cost-effectiveness ratio (ICER) of $40.3 as seen in Table 7. The cost-effectiveness ratio for the standard and the new intervention was $472.2 and $170.4 respectively per referral completed. This shows that the EmTS is more cost-effective when compared to the standard as it gives a lower cost-effectiveness ratio. Finally, both the costs and effectiveness were discounted at 5%. The total discounted costs for standard and new interventions were $133,679.4 and $160,148.8 respectively. The discounted effectiveness was $283 and $940 for standard and new interventions respectively.

**Table 7:** Cost–effectiveness ranking.

Cost-Effectiveness Rankings Report						
Strategy	Cost ($)	Incr Cost* ($)	Eff**	Incr Eff	C/E	ICER
Standard referral system (Ambulance only)	140,715	27,862	298		472.2	
Emergency Transport System (Ambulance plus community Taxi)	168,578	657	989	691	170.4	40.3

Incr Cost: Incremental Cost; Eff: number of completed referrals; Incr Eff: Incremental cost–effectiveness; C/E: Cost–effectiveness ratio; ICER: Incremental cost–effectiveness ratio

## Discussion

The EmTS designed and implemented by the m-mama program was a more cost-effective intervention in addressing emergency referrals compared to the standard emergency referral system in Shinyanga region. This innovative system costs $170.4 per completed referral as compared to $472 per referral by using ambulances alone. The program data revealed that the EmTS was used more in the rural areas where ambulances have limited functionality given the remoteness of the areas. To this end therefore, the use was less in urbanized areas of Shinyanga MC compared to the district councils which were predominantly rural areas.

Evidence presented in this evaluation were comparable to results from other contexts. For example, emergency referral system using ambulances implemented in Burundi by Médecins sans Frontières (MSF) reported the cost per referral to be £61 [19] equivalent to $121 per completed referral when accounted for annual inflation rate [20]. These costs are slightly lower than the costs of the m-mama EmTS per referral, but four times lower compared to the Tanzania ambulance cost per referral indicating the need for Tanzania to consider scaling up the m-mama EmTS. This difference can be attributed to the difference in health system structure and governance between Tanzania and Burundi and their economic levels. Moreover, in comparison with another local contexts, the cost per ambulance referral $472 reported in this study is higher compared to that reported for ambulance referrals in handling surgical emergencies conducted in six district hospitals found in Arusha and Kilimanjaro regions of Tanzania [21]. The study reported the ambulance cost per referral for surgical emergencies to range from $19 for Meru district hospital to $208 for Huruma district hospital [21]. This indicates a regional variability of ambulance costs per referral. In the same study, higher costs for ambulances than those reported in this study were reported for two district hospitals in Zambia which are $538 for Namwala district hospital and $534 Siavonga district hospital [21].

Ambulances are an important part of the emergency transportation systems [22, 23]. However, they have been criticized their high operational costs when patients have no other means of transportation during emergency situations [24]. The costs and operational challenges have led to interest in the potential of mHealth based interventions. mHealth-based interventions are likely to be acceptable among healthcare stakeholders in handling maternal emergency referrals [25]. Moreover, evidence has shown that the use of mHealth interventions in emergency services led to reduced per-capita ambulance volume by at least 6.7% [26]. Digitalizing emergency transportation system and adding community-based means alongside standard ambulances could help elevate the effectiveness of emergency transportation systems in resource limited settings.

Cost-effective transportation interventions for maternal care are an important part of the country’s strategy to reduce maternal mortality [27]. They can address the delay in reaching the health facility which is one of the key drivers of maternal mortality in Low and Middle-Income Countries including Tanzania [28]. Emergency maternal transportation interventions can be more effective if integrated within a referral system or as an additional strategic intervention aimed at improving the access to healthcare service delivery points present [29].

Lack of cost-effectiveness evidence of interventions make their scalability questionable [29]. This study has presented cost-effectiveness analysis using number of completed referrals as a proxy for effectiveness of an innovative emergency transport system aimed to address delay in reaching the health facility during maternal emergencies in Tanzania. This analysis has shown EmTS system to be cost effective in Shinyanga region, further studies in the scale-up phase are warranted to generate generalizable results for Tanzania. Similarly, in other settings where new interventions are being implemented, it is commendable to integrate cost effectiveness analysis studies from the planning phase so that appropriate cost and effectiveness data can be captured throughout the program implementation [30]. This will generate robust evidence for program feasibility, scale up and sustainability [31]. A costing framework proposed by Sohn et al can be used to help inform the design of costing the implementation of public health interventions [31].

Evidence presented in this study should be interpreted in line with some limitations. We used the data for only one-year FY2020/2021, which was suitable for the current analysis. Moreover, because we used the data from only one region, we acknowledge regional variability which needs to be considered for generalizable evidence. Sensitivity analysis of cost-effectiveness analysis was not done owing to lack of enough data and therefore the findings cannot be generalized with certainty to other regions and contexts. In this study, a proxy measure of effectiveness, which is the number of completed referrals, was used. This does directly reflect effectiveness in terms of health outcomes and therefore, efforts to scale-up the intervention should consider adding more strong measures of outcome such as DALYs or QALYs. Retrospective analysis of cost data could have resulted in the underestimation of the cost for implementing the EmTS intervention [31]. Despite the limitations, this is the first study in Tanzania to examine cost effectiveness of a community-based innovative intervention aiming at combating the second maternal delay, an important cause of maternal mortality in Tanzania, and other areas with similar context.

## Conclusion and recommendation

The introduction of m-mama emergency transportation system has shown to be cost effective in completing emergency referrals compared to the use of ambulance alone in Shinyanga region. Given the high burden of maternal mortality in Tanzania, efforts to scale-up the program to other regions in Tanzania should consider taking a phasic approach and, in each phase, generating more cost-effectiveness evidence upon implementing the intervention in other regions. Importantly, Implementation science methods should be used to document and use the lessons to improve the effectiveness of the EmTS during the scale up phase.
